# Ultrasensitive Chemical Analysis on Gold Nano Popcorn Substrate Using Digital Surface-Enhanced Raman Scattering

**DOI:** 10.3390/molecules30061371

**Published:** 2025-03-19

**Authors:** Soyeon Lee, Jaebum Choo

**Affiliations:** Department of Chemistry, Chung-Ang University, Seoul 06974, Republic of Korea; dearosie@naver.com

**Keywords:** digital SERS, Au nano popcorn substrate, surface-enhanced Raman scattering, digital analysis, ultrasensitive chemical analysis

## Abstract

This study presents a digital surface-enhanced Raman scattering (SERS) method to enhance the sensitivity of SERS detection for low-concentration analytes. Conventional SERS analysis using average Raman intensity faces limitations in distinguishing low concentrations due to the substrate’s sparse distribution of target molecules. To overcome this challenge, we used a binary code-based data analysis approach. Gold nano popcorn substrates were utilized for high-sensitivity detection, with malachite green isothiocyanate (MGITC) as the target molecule. Raman mapping data were analyzed using both the conventional average Raman intensity method and the proposed digital SERS approach. In the digital SERS method, a threshold value was established based on the mean and standard deviation of Raman signals in the absence of target molecules. Pixels with Raman intensities exceeding this threshold were assigned a value of “1”, while those below were assigned “0”. Quantification was then performed based on these digital counts corresponding to MGITC concentrations. Our results demonstrate that the digital SERS method significantly improved the ability to distinguish and quantify analytes in low-concentration ranges that were indiscernible using the conventional approach. This analytical technique shows promise for ultrasensitive chemical analysis and expands the capabilities of SERS-based detection methods, potentially revolutionizing the field of trace analyte detection.

## 1. Introduction

Surface-enhanced Raman scattering (SERS) has emerged as a powerful analytical technique capable of detecting molecules at ultra-low concentrations, even down to single-molecule levels [1,2,3]. This remarkable sensitivity is achieved by amplifying Raman scattering signals through localized surface plasmon resonance (LSPR) occurring on metal surfaces or nanostructures [4,5,6,7]. The widespread applications of SERS in fields such as disease diagnosis, chemical analysis, and nanotechnology underscore its significance in modern scientific research [8,9,10,11,12]. Despite its potential, SERS faces challenges in signal sensitivity and reproducibility, particularly at very low analyte concentrations. These limitations stem from the uneven distribution of “hot spots”—areas of intense electromagnetic field enhancement that account for a disproportionate amount of the total Raman signal [13,14]. As analyte concentration decreases, the contribution of SERS signals to the overall signal diminishes, leading to increased fluctuations and reduced detection sensitivity.

To address these challenges, researchers have primarily focused on developing plasmonic nanostructures with enhanced amplification effects [15,16,17,18,19,20]. However, this approach alone may not fully resolve the issues of reproducibility and sensitivity at extremely low concentrations. In response to these limitations, a new data processing method called digital SERS was proposed [21,22]. The digital SERS method divides the analysis area into micro-compartments and digitizes each compartment for data processing. Recently, a Au nano popcorn substrate with highly uniform nanogaps has been developed by growing gold particles on Au nano dimple substrates [23]. This study aims to implement reproducible high-sensitivity SERS detection by introducing the digital SERS technique to these Au nano popcorn structures. This approach is particularly effective for very low analyte concentrations, where traditional methods struggle to provide reliable results.

To demonstrate the efficacy of the digital SERS method, we used malachite green isothiocyanate (MGITC), widely used as a Raman reporter in SERS detection, as a model analyte. Raman mapping images were obtained by loading different concentrations of MGITC onto the Au nano popcorn substrates and continuously measuring Raman signals over specific areas. We then analyzed the Raman mapping data using both conventional methods and our proposed digital SERS approach. In the conventional SERS analysis, characteristic Raman peak intensities for each pixel were averaged to calculate quantitative values. For the digital SERS method, a threshold value based on the measured Raman peak intensities for the digital SERS method was established. Signals above this threshold were assigned a value of “1”, while those below were assigned “0”. These binary values were then decoded into black and white colors for analysis [24,25]. The primary objective of this research is to demonstrate the potential of digital SERS detection technology in overcoming the limitations of reproducibility and sensitivity inherent in conventional SERS. By introducing this novel data processing approach, we aim to expand the capabilities of SERS-based detection methods and pave the way for more reliable and sensitive analytical techniques in various scientific disciplines.

## 2. Results and Discussion

**Figure 1:**
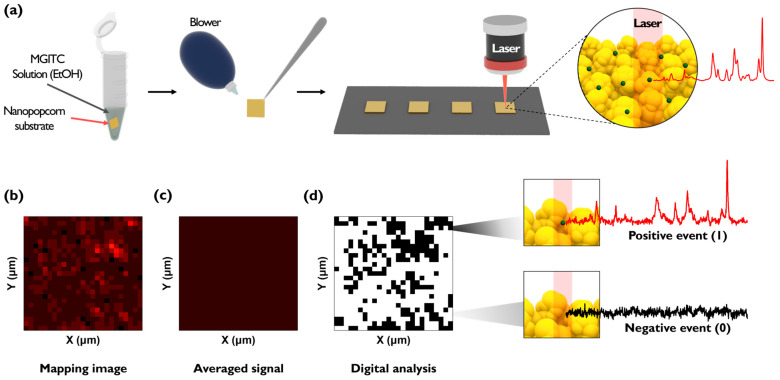
Illustration of digital SERS. (**a**) Experimental procedure to obtain Raman mapping image of MGITC on Au nano popcorn substrates. (**b**) SERS mapping image obtained from raw Raman mapping data. (**c**) Raman image using average intensity for all pixels of Au nano popcorn substrate. (**d**) Digital decoding SERS image.

The Au nano popcorn substrate used in the experiment was fabricated based on previous papers reported by our research group [26,27,28,29]. The Au nano popcorn substrate has uniform nanogaps on its surface, creating numerous hot spots where Raman signals are greatly amplified due to LSPR when analytes are positioned on the structure’s surface. The enhancement factor (EF) of the Au nano popcorn substrate, calculated by comparing the Raman signal of MGITC measured on a glass substrate, was evaluated to be 7.18 × 10^7^. Therefore, the Au nano popcorn substrate enables high-sensitivity detection of target molecules. The overall experimental process is illustrated in Figure 1a. A 3 × 3 μm^2^ piece of Au nano popcorn substrate was placed in a 1.6 mL microtube, and 200 μL of MGITC solution diluted with ethanol was added. After reacting for 1 h at 500 rpm, the remaining solution was removed using a blower. After washing, the nano popcorn substrate was fixed on a slide glass, and Raman signals were measured. Figure 1b shows a conceptual diagram of the Raman mapping image measurement converted to digital code after acquiring the Raman signal after loading MGITC. Figure 1b displays the Raman mapping image obtained by monitoring the intensity change in MGITC’s characteristic Raman peak at 1614 cm^−1^. In the conventional data analysis, the average value of Raman peak intensities is correlated with the concentration. Herein, Raman peak intensities measured at all pixels are averaged to analyze the results (Figure 1c). In contrast, digital SERS sets a threshold value and converts the Raman intensity of each pixel into a digital code for analysis. If a pixel’s Raman intensity exceeds the threshold value, it is considered a “1” or positive event and marked in black. If it does not exceed the threshold value, it is regarded as a “0” or negative event and marked in white (Figure 1d). A “1” indicates that a SERS intensity that is distinguishable from the background signal was obtained, while a “0” means it cannot be distinguished from the background signal.

**Figure 2:**
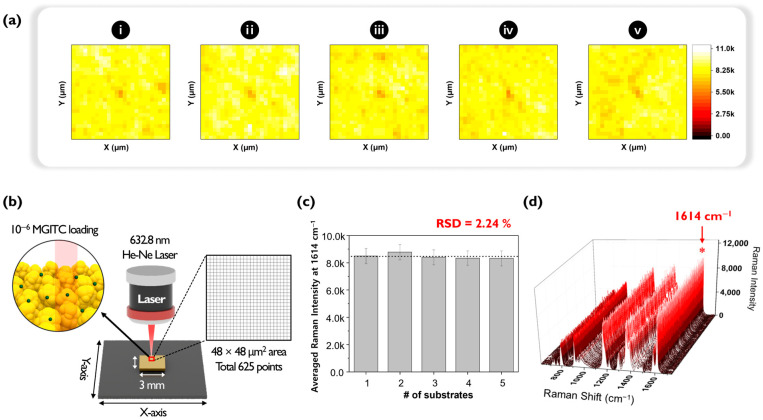
(**a**) Raman mapping images of MGITC for five different nano popcorn substrates (**i**~**v**). (**b**) Raman mapping method for Au nano popcorn substrate. The total area of each Au nano popcorn substrate was scanned over an area of 48 × 48 μm^2^ range with 2 × 2 μm^2^ mapping steps, totaling 625 pixels. The samples are exposed to 10% laser power with 0.1 s exposure time. (**c**) Relative standard deviation (RSD) to demonstrate substrate-to-substrate fluctuations. (**d**) Raman spectral data obtained from all mapping points in substrate to demonstrate spot-to-spot fluctuations.

An experiment was conducted to evaluate the reproducibility of Au nano popcorn substrates by assessing the fluctuation in Raman signal intensity between different substrates. As shown in Figure 2, five distinct Au nano popcorn substrates were used for this purpose. For quantitative experiments at ultra-low analyte concentrations, it is crucial that the fluctuation between different substrates is minimal, and that the Raman intensity remains consistent. First, five Au nano popcorn substrates were cut to identical sizes. Second, concentration and other reaction conditions were kept constant for all substrates. Third, using a computer-controlled x-y transitional stage, a 48 μm × 48 μm area was scanned with a 2 μm step size, resulting in a 625-pixel image (Figure 2b). Finally, Raman mapping images were obtained by color decoding the Raman intensity at 1614 cm^−1^, characteristic of the MGITC molecule. Figure 2a shows the five Raman mapping images obtained from this experimental process. A color scale bar on the right side of the image indicates different Raman intensities. The uniformity of pixel colors in these images signifies the homogeneity of the plasmonic substrate, which minimizes substrate-to-substrate intensity fluctuations and increases reproducibility. Figure 2c compares each substrate’s average Raman peak intensities in a bar graph. The relative standard deviation (RSD) of the Raman peak intensity at 1614 cm^−1^ for five substrates was calculated to be 2.24%, indicating excellent reproducibility of the plasmonic substrates used in this study. Figure 2d plots all 625 Raman spectra for easy comparison. The RSD calculated from the characteristic Raman peak intensity of MGITC at 1614 cm^−1^ was 6.54%, demonstrating outstanding pixel-to-pixel reproducibility within the substrate. Therefore, it could be concluded that the nano popcorn substrate used in this study proved highly suitable for ultra-low concentration quantitative analysis using the digital SERS method due to its excellent reproducibility between substrates as well as between spots in a single substrate.

**Figure 3:**
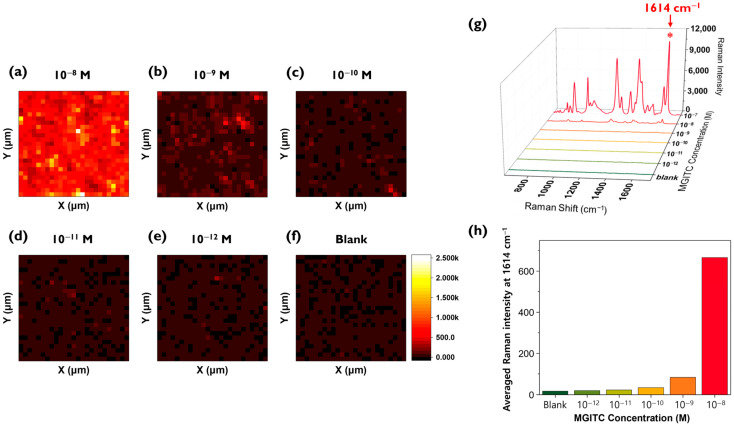
(**a**–**f**) Raman mapping images of MGITC in the 0 to 10^−8^ M range using the characteristic Raman peak intensity at 1614 cm^−1^. A 48 μm × 48 μm area was mapped with a 2 μm step size, resulting in Raman data for a total of 625 pixels. (**g**) Average Raman spectra of 625 pixels for each concentration. (**h**) Histogram of the average Raman peak intensity in the 0 to 10^−8^ M range.

After completing the reproducibility assessment, MGITC solutions were diluted to various concentrations and loaded onto the Au nano popcorn substrate to measure Raman peak intensities. Color decoding was performed using the characteristic Raman peak intensity at 1614 cm^−1^ of MGITC (Figure 3a–f). A 48 μm × 48 μm area was mapped with a 2 μm step size, resulting in Raman data for a total of 625 pixels. The reason for choosing a 2 μm × 2 μm pixel size for the mapping image is due to the approximate laser beam size. The Raman mapping measurements used a 20× magnification/NA 0.4 objective and a 633 nm He-Ne laser, which produces a focused laser beam size of 1.93 μm. The step size (2 μm) was set similarly to the laser beam size to achieve high spatial resolution. When measuring the Raman mapping images using this method, it was observed that the overall Raman mapping image became darker as the concentration of the MGITC solution decreased. Figure 3g shows the average Raman spectra of 625 pixels for each concentration. As the concentration of MGITC decreases, the intensity of the characteristic Raman peak at 1614 cm^−1^ also decreases. Since it is difficult to discern the low concentration range (below 10^−9^ M) in this figure, a histogram was created using only the Raman peak intensities at 1614 cm^−1^ for the low concentration range (10^−8^ M to blank) (Figure 3h). This graph shows that even in the low concentration range, the average Raman peak intensity gradually decreases as the concentration decreases, but it is not easy to distinguish the Raman peak intensity at concentrations below 10^−10^ M using this conventional SERS mapping method.

**Figure 4:**
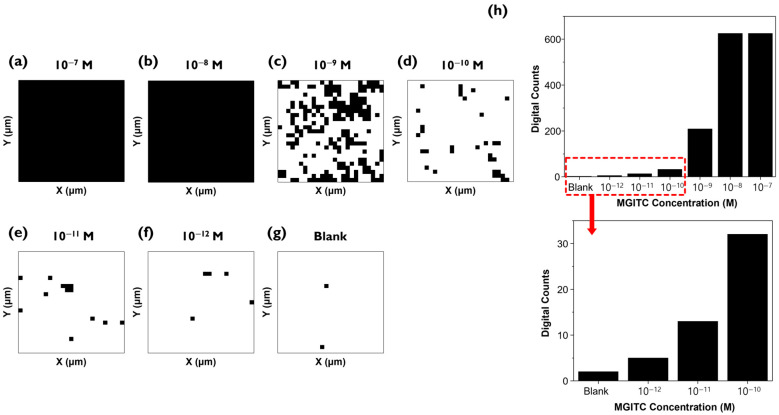
(**a**–**g**) Digital SERS mapping image of MGITC on Au nano popcorn substrate in the 0 to 10^−7^ M range. (**h**) Histogram of the number of digital counts (“1”) in the 0 to 10^−7^ M range (**top**). Enlarged histogram for the low concentration range, 0 to 10^−10^ M (**bottom**).

The Raman mapping data measured in Figure 3 were analyzed using the digital SERS technique (Figure 4a–g). The average Raman peak intensity and standard deviation of a blank sample without the analyte MGITC determined the threshold value. The threshold was set to minimize the influence of background noise signals so that over 99% of the 625 pixels in the blank fell within this range [21,22]. This method is identical to determining the limit of detection (LOD) in conventional bioassays [27,28]. While traditional LOD determination methods use the average and standard deviation of intensities for each concentration to plot a graph and then determine the LOD using the blank sample’s average intensity + 3× standard deviation formula, digital SERS distinguishes between positive and negative for each pixel in the mapping image based on the threshold value, without calculating the average Raman peak intensity. As seen in Figure 3a–g, average Raman intensity values from SERS data can accurately analyze Raman data for high concentrations, but there are limitations in distinguishing low-concentration samples. Because many analyte molecules exist on the nanostructured surface, it is possible to distinguish high-concentration samples. In the case of low-concentration samples, enhanced Raman signals appear only in extremely limited areas at low concentrations because the analyte molecules are not uniformly distributed on the nanostructured surface and rarely exist at hotspots [14]. However, when analyzing very low analyte concentrations using conventional SERS, the SERS intensity from hotspots contributes little to the overall signal intensity, making it difficult to distinguish from background noise signals and reducing sensitivity in the quantitative analysis due to increased Raman signal fluctuation.

To solve this problem, digital SERS technology can be introduced, which defines cases exceeding the threshold value for Raman peak intensity of each pixel as “1”, and those that do not as “0”, then counts the number of “1”s. This method makes accurate quantitative analysis possible even when the distribution of molecules on the Au nano popcorn substrate is not uniform in the low concentration range. As seen in Figure 4a, at high concentrations, MGITC molecules are distributed across the entire substrate, showing a signal of 1 in all pixels (Figure 4a,b). In the low concentration range, which conventional methods could not distinguish, the number of black pixels gradually decreases as the concentration decreases (Figure 4c–e). Figure 4h shows the count of black pixels from Figure 4a (top). The low concentration range of 10^−10^ M to 10^−12^ M and the blank are enlarged as they are difficult to discern visually (bottom). This figure confirms the trend of decreasing digital counts as the concentration decreases.

**Figure 5:**
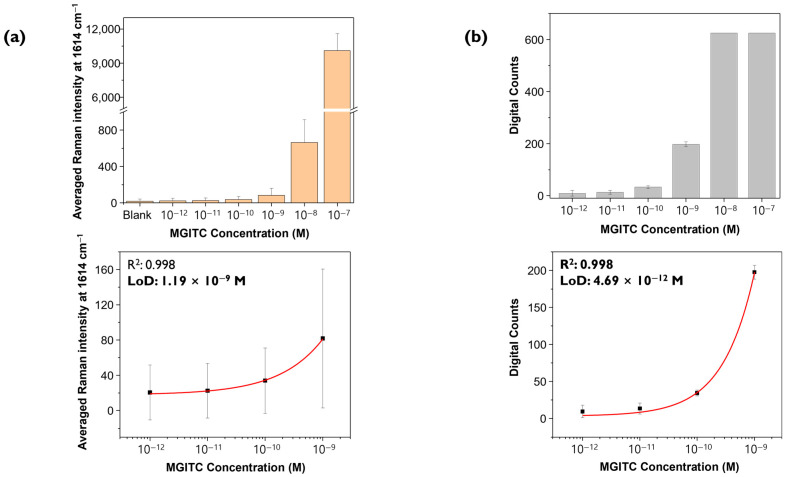
(**a**) Histogram of the average Raman peak intensity at 1614 cm^−1^ (**top**) and corresponding calibration curve (**bottom**). (**b**) Histogram of the number of digital counts (**top**) and corresponding calibration curve (**bottom**). Error bars indicate the standard deviations from three measurements.

Raman spectra were measured three times for the same concentrations of MGITC sample under identical measurement conditions (633 nm laser, power 10% = 0.8 mW, exposure time 1 s, objective lens 20×, 48 μm × 48 μm, 2 μm step size). Three Raman mapping images, each consisting of 625 pixels, were obtained and analyzed using the conventional SERS mapping and digital SERS methods to obtain corresponding calibration curves. Figure 5a shows a histogram for the average SERS peak intensity at 1614 cm^−1^ for 625 points measured three times for each concentration. The *y*-axis represents the average Raman peak intensity, and the error bars indicate the standard deviations for each concentration. Using a 4-parameter fitting equation, the calibration curve was determined and yielded an R^2^ value of 0.998, and the calculated LOD was 1.19 nM. Figure 5b shows the results of analyzing the same Raman spectral data using digital decoding. As seen in Figure 4a, for relatively high concentrations such as 10^−7^ M and 10^−8^ M, Raman intensity values for all pixels are saturated, resulting in a positive event (“1”) for all pixels, making it impossible to determine quantitative relationships. Therefore, the histogram shows the change in digital counts according to the change in low MGITC concentrations below 10^−9^ M. The average value and standard deviation of digital counts were calculated from images obtained by mapping three times for each concentration. The threshold value was set as the LOD in the blank sample, which is the theoretical LOD of conventional SERS, as shown in Figure 3a. The average value of digital counts is represented as the *y*-value, and the standard deviation is represented as error bars. Similarly, using the 4-parameter fitting, the quantification curve yielded an R^2^ value of 0.998, and the calculated LOD was 4.69 pM, about 300 times lower than the conventional SERS method. The R^2^ values of both graphs (≥0.99) confirm that the quantification curves were determined meaningfully and reliably. As expected, the digital SERS method allowed for accurate quantitative analysis at lower concentrations than the conventional SERS method. According to Figure 5b, the LOD for digital SERS was determined to be 4.69 pM, which is included in the concentration range of the quantitative curve. However, evaluating the usefulness of the digital SERS analysis method based on Raman spectral data for only four MGITC concentrations in the range below 10^−9^ appears to be of little significance. Therefore, additional Raman measurements for more concentrations were necessary for more reliable quantitative analysis.

**Figure 6:**
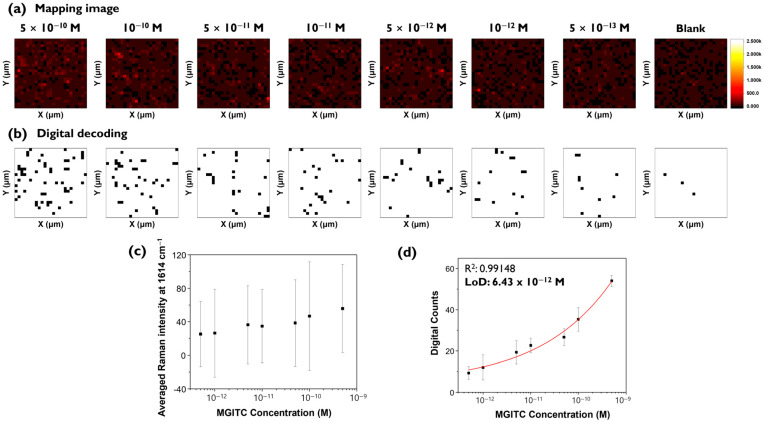
(**a**) Raman mapping and (**b**) digital decoding images of MGITC in the 0 to 5 × 10^−10^ M range. Corresponding calibration curves for (**c**) Raman mapping and (**d**) digital decoding images. Error bars indicate the standard deviations from three measurements.

To demonstrate that the digital SERS method can analyze target analytes at low concentrations more accurately, experiments were conducted at more finely divided intervals at lower concentrations from 5 × 10^−10^ M to 5 × 10^−13^ M (Figure 6). Figure 6a shows conventional Raman mapping images for additional MGITC concentrations, and Figure 6b shows corresponding digital SERS images. The scale bar for the Raman mapping image was set the same as in Figure 3a. Figure 6a shows that it is difficult to distinguish between the color-decoding Raman mapping images for all concentration ranges. In contrast, the binarized images using digital SERS could be visually distinguished for all images as shown in Figure 6b. Figure 6c,d compares the calibration curves for conventional SERS and digital SERS. For conventional SERS, a clear decrease in Raman intensity with concentration was not observed, and there was a large standard deviation and loss of linearity. In contrast, for digital SERS, the number of pixels showing “1” changed significantly with concentration changes. Only for digital SERS, it was possible to determine a meaningful quantitative curve, with an R^2^ value of 0.991 and a LOD of 6.43 pM (Figure 6d). The LOD for digital SERS in this experiment was similar to the previous experimental result determined for smaller concentrations (Figure 5b). A significant reduction in error bar fluctuation was also observed for digital SERS. These results confirmed that digital SERS can achieve higher sensitivity and lower LOD values than conventional methods.

## 3. Materials and Methods

Ethanol (99.5%) and tetrahydrofuran (THF) was purchased from Sigma-Aldrich (St. Louis, MO, USA). Malachite green isothiocyanate (MGITC) was purchased from Invitrogen Corporation (Carlsbad, CA, USA). Ultrapure water (0.055 μs/cmc) was obtained from a Laboratory Water System (Göttingen, Germany). A 125 μm thick polyethylene naphthalate (PEN) polymer substrate (Dupont, Wilmington, DE, USA) was used after removing the protective film. A polymer nano dimple pattern was fabricated on the PEN film by O_2_ ion beam bombardment in a linear moving substrate. A linear O_2_ ion beam with a width of 300 mm was generated using a linear ion source [30]. An O_2_ flow rate was 70 sccm with the vacuum process chamber pressure of 0.9 mTorr. The PEN substrates were reciprocated at a linear moving speed of 10 mm s^−1^, followed by 60 scans. The ion dose per scan was 2.3 ± 0.2 × 10^15^ cm^−2^ using a Faraday cup that reduced secondary electron emission by magnetic fields. The mean ion energy was 700 ± 70 eV measured with an ion energy analyzer [31]. A 100 nm thick Au layer was deposited directly on PEN nano dimples at a deposition rate of 2.0 Å s^−1^ using a thermal evaporation system (LAT, Osan, Republic of Korea). The base pressure of the chamber was 9.6 × 10^−6^ Torr. Then, the prepared Au/PEN nano-dimple substrates were processed with 97% perfluorodecanethiol (Sigma-Aldrich, St. Louis, MO, USA). A total of 10 μL of 97% perfluorodecanethiol solution was poured into a glass Petri dish, the Petri dish lid was put to the Au/PEN nano dimple substrate, and the lid was closed for 2 h [23]. Next, an 80 nm thick Au layer was deposited onto the PFDT-treated Au/PEN nano dimple substrate at a deposition rate of 0.3 Ås^−1^ through a thermal evaporation process (LAT, Osan, Republic of Korea). The base pressure of the chamber was 9.6 × 10^−6^ Torr. The deposition rate was watched using a quartz crystal microbalance.

Before the experiment, the substrate was cut into 3 × 3 μm^2^ size and washed with tetrahydrofuran and distilled water. Then, MGITC solution was diluted to the concentration using pure ethanol as buffer (10^−7^ M~5 × 10^−13^ M, blank). The cut substrate was added to 200 μL different concentrations of MGITC solution and reacted by shaking (500 rpm) for 1 h. After the reaction, the substrate was added to 200 μL of DW, washed by shaking for 5 min, and fixed on a slide glass to dry. To evaluate reproducibility between substrates, five Au nano popcorn substrates of 3 × 3 μm^2^ size were prepared and reacted in 10^−6^ M of MGITC solution for 1 h.

The SERS spectra and Raman mapping images were obtained by an inVia Renishaw Raman microscope system (Renishaw, New Mills, UK). A He-Ne laser operating at 632.8 nm was used as the excitation source. Raman mapping images were obtained using a 20× objective lens and measured with an exposure time of 1 s and a laser power of 10%. To evaluate reproducibility, Raman mapping images were obtained using a 20× objective lens and measured with an exposure time of 0.1 s and laser power of 10%. The characteristic Raman peak of MGITC at 1614 cm^−1^ was scanned over an area of 48 × 48 μm^2^ range with 2 × 2 μm^2^ mapping steps, for a total of 625 pixels. The baseline correction of Raman spectra was carried out using WiRE V 5.3 software (Renishaw, Newmills, UK). Spectral analysis and Raman mapping image, digital decoding image generation were performed using Origin 2017 64Bit software (OriginLab Corporation, Northampton, MA, USA).

The data were processed using the mean and standard deviation of pixels. An area of 48 × 48 μm^2^ range was measured three times at 2 μm step size, yielding a total of 1875 points of data. For data processing, we acquired the mean and standard deviation of the 1875 points to obtain a calibration curve. Based on the mean and standard deviation of the intensity of the blank (without analyte), the mean + 3 × standard deviation was set as the threshold value, which was set to “0” if it was less than the threshold value and “1” if it was greater than the threshold value. For each concentration, the number of pixels higher than the threshold value was counted and calculated for each concentration.

## 4. Conclusions

Recently, the SERS technique is gaining popularity as an analytical technique due to its high sensitivity, quantitative analysis, multiplexed detection, and rapid detection capabilities. SERS allows for analysis of trace amounts of substances, even down to a single molecular level. However, when the concentration of the analyte becomes extremely low, the signal-to-noise ratio decreases dramatically due to the influence of background noise, making it difficult to distinguish SERS signals generated from the plasmonic substrate’s hotspots from noise. In this study, Raman reporter molecule MGITC was loaded at different concentrations onto Au nano popcorn substrates previously developed by our research group. The resulting Raman mapping images were analyzed using both conventional SERS and digital SERS methods. The conventional SERS method involves color decoding of Raman peak intensities for each pixel. However, at low concentrations, the signal-to-noise ratio decreases, making it difficult to distinguish color changes corresponding to concentration. Consequently, quantitative analysis using spectral data in conventional SERS results in increased error deviation, reducing accuracy, sensitivity, and reproducibility. The LOD for MGITC determined by conventional SERS was at the nM level, with concentrations below this becoming impossible to quantify due to excessive error fluctuations.

To address this issue, we introduced the digital SERS method. This approach involves setting a threshold value for Raman peak intensity, defining pixels as “1” if above the threshold and “0” if below, and transforming Raman mapping images into digital Raman images for analysis. This method allows for accurate quantitative analysis by counting the number of pixels showing “1”, even when low-concentration MGITC molecules are unevenly distributed on the substrate surface. The introduction of the digital SERS method enabled accurate quantitative analysis for MGITC concentrations below 10^−9^ M by reducing error fluctuations. The LOD was significantly improved to the pM range, showing greater sensitivity compared to the conventional SERS method. Analysis of the same Raman mapping data yielded an LOD of 1.19 nM using conventional SERS and 4.69 pM using digital SERS. Since improving sensitivity through experimental methods alone is very challenging, it is expected that complementary use of the digital SERS analysis method can greatly enhance the sensitivity and reproducibility of analyte detection.

## Data Availability

The original contributions presented in this study are included in the article, further inquiries can be directed to the corresponding author.

## Abbreviations

The following abbreviations are used in this manuscript:

SERS	Surface-enhanced Raman scattering
MGITC	Malachite green isothiocyanate
LSPR	Localized surface plasmon resonance
